# Measurement of the emission spectrum of a semiconductor laser using laser-feedback interferometry

**DOI:** 10.1038/s41598-017-07432-0

**Published:** 2017-08-03

**Authors:** James Keeley, Joshua Freeman, Karl Bertling, Yah Leng Lim, Reshma A. Mohandas, Thomas Taimre, Lianhe H. Li, Dragan Indjin, Aleksandar D. Rakić, Edmund H. Linfield, A. Giles Davies, Paul Dean

**Affiliations:** 1 School of Electronic and Electrical Engineering, University of Leeds, Leeds, LS2 9JT UK; 2 School of Information Technology and Electrical Engineering, The University of Queensland, St Lucia, QLD 4072 Australia; 3 School of Mathematics and Physics, The University of Queensland, St Lucia, QLD 4072 Australia

## Abstract

The effects of optical feedback (OF) in lasers have been observed since the early days of laser development. While OF can result in undesirable and unpredictable operation in laser systems, it can also cause measurable perturbations to the operating parameters, which can be harnessed for metrological purposes. In this work we exploit this ‘self-mixing’ effect to infer the emission spectrum of a semiconductor laser using a laser-feedback interferometer, in which the terminal voltage of the laser is used to coherently sample the reinjected field. We demonstrate this approach using a terahertz frequency quantum cascade laser operating in both single- and multiple-longitudinal mode regimes, and are able to resolve spectral features not reliably resolved using traditional Fourier transform spectroscopy. We also investigate quantitatively the frequency perturbation of individual laser modes under OF, and find excellent agreement with predictions of the excess phase equation central to the theory of lasers under OF.

## Introduction

Optical feedback (OF) occurs when radiation emitted from a laser is reflected by an external target and partially reinjected into the laser cavity^1, 2^. The effects of OF have been observed since the early days of laser development^3^, and are well known to induce undesirable phenomena in lasers including increased intensity noise^4^, coherence collapse^5^, chaotic behaviour^6^ and transitions between laser operating regimes^7^. Nevertheless, the optical (homodyne) mixing that occurs between the reinjected radiation and the intra-cavity photon field can also cause predictable and measurable perturbations of the laser operating parameters that depend on both the phase and amplitude of the reinjected field^1, 2^. The laser response to OF, which in the case of semiconductor lasers can be sensed via the laser terminal voltage^8^, thus encapsulates information about the external cavity and the optical properties of the target. This has led to the concept of laser-feedback interferometry (LFI)^9^, utilising the self-mixing effect, in which a single laser device acts as a source, local oscillator, mixer, and shot noise-limited detector, thus forming a compact interferometric sensor. The experimentally simple nature of such schemes has motivated their use in a range of sensing applications^9^ including coherent imaging^10, 11^ and microscopy^12, 13^, distance-ranging^14^, vibrometry^15^ and displacement sensing^16^, materials analysis^17, 18^, and Doppler flow measurements^19^. Moreover, the applicability of LFI has been demonstrated across a wide range of class-A and class-B laser systems spanning from the visible to microwave region, and including gas lasers^20^, in-plane semiconductor diode lasers^21^, vertical-cavity surface emitting lasers (VCSELs)^21, 22^, mid-infrared^23^ and terahertz (THz)-frequency^11, 18, 24, 25^ quantum cascade lasers (QCLs), interband cascade lasers^26^, fiber^27^ and fiber ring lasers^28^, and solid-state lasers^29^. This remarkable universality of the self-mixing phenomenon and its intrinsic dependence on the electronic and optical properties of lasers has also enabled measurement of fundamental laser parameters, including the linewidth enhancement factor (LEF)^30, 31^ and laser linewidth^32, 33^.

In this work we demonstrate a new modality of LFI in which changes to the terminal voltage of a THz QCL in response to an extension of the external cavity are used to infer the emission spectrum of the solitary laser, for both single- and multiple-longitudinal mode operating regimes. Whilst applicable across all types of semiconductor lasers, our scheme offers a simple alternative to Fourier transform infrared (FTIR) spectroscopy approaches typically employed for spectral characterisation of mid- and far-infrared lasers, and furthermore avoids the reliance on slow, insensitive or cryogenically-cooled THz detectors. Central to our approach is the ability to recover interferometric voltage signals with a high signal-to-noise ratio in the regime of weak OF; in such regimes the spectral characteristics of the solitary laser dominate over those of the external cavity, supressing phenomena such as line splitting and mode hopping^7, 34^. This is made possible by the homodyne nature of the LFI scheme, which inherently provides very high sensitivity, potentially at the quantum noise limit^35^. We also measure quantitatively, for the first time to our knowledge, the frequency change of solitary laser modes under OF. We confirm the observation of small perturbations to the solitary laser emission frequency, and find excellent agreement with predictions of the excess phase equation central to the theory of lasers under OF.

## Results

### Spectral characterisation by laser-feedback interferometry

**Figure 1:**
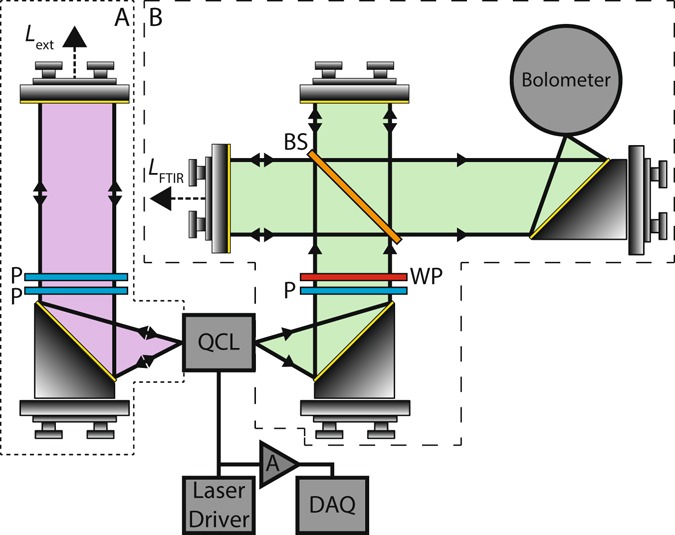
Schematic diagram of the experimental system showing laser-feedback interferometer (box A) and Fourier transform infrared (FTIR) spectrometer (box B), used for the purposes of spectral comparison. Wire grid polarisers (P) are employed to control the level of optical feedback in the laser-feedback interferometer. A quarter-wave plate (WP) and grid polariser provide optical isolation in the FTIR spectrometer. BS‒silicon beam splitter; DAQ‒digital acquisition board; A-Amplifier.

In our experiment a QCL emitting at ~2.25 THz was used as the laser source (see Methods). Radiation from the front facet of the device was collimated and reflected back to the laser cavity using a planar mirror, as shown in Fig. 1 (box A). Two wire grid polarisers were positioned in the external cavity to provide control over the level of OF to the laser; rotation of the second of these polarisers through an angle *θ* relative to the axis of the first results in a power attenuation factor of cos^4^ *θ* in double-pass, enabling a range of feedback parameters to be achieved from *C* ≈ 0 (no feedback) to *C* ≈ 1.5. To perform spectral analysis using LFI the feedback parameter was set to *C* ≈ 0.6, ensuring operation in the regime of weak feedback such that the spectral characteristics of the solitary laser dominate over those of the external cavity.

**Figure 2:**
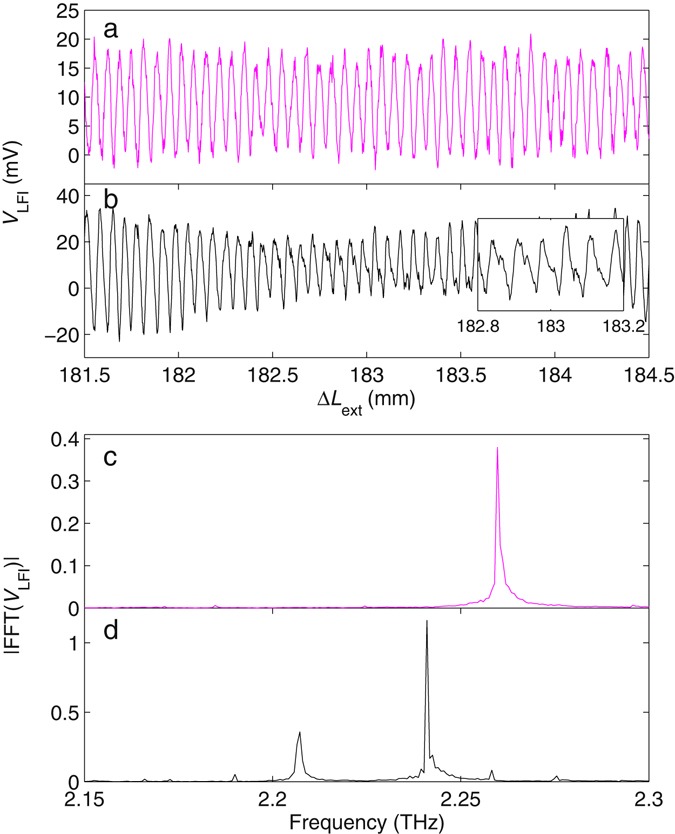
Interferograms recorded via the laser terminal voltage *V*_LFI_ with the laser operating on (**a**) single and (**b**) multiple longitudinal cavity modes. Voltage signals have been amplified by a 22 dB differential amplifier. (**c**) and (**d**) show the respective emission spectra obtained by performing a fast Fourier transform of the complete interferograms corresponding to data presented in (**a**) and (**b**).

Figure 2(a) shows an exemplar interferogram recorded via the laser terminal voltage (see Methods), with the laser emitting in a single longitudinal mode, achieved at a dc drive current of *I*_*d*_ = 725 mA. In this case the LFI voltage signal *V*_LFI_ is described well by a rate equation reduction of the complex field and carrier density of lasers under OF, as first presented in the seminal work of Lang and Kobayashi (L–K)^36^. This deterministic formalism describes how changes in the intracavity field amplitude under OF cause perturbation to the carrier density, which in turn modifies the instantaneous frequency of the laser. In the steady-state condition this model can be shown to reduce to a set of equations for the laser frequency and the threshold carrier density under OF (see Methods). The latter of these equations [Eq. (2)] relates directly to the observable LFI voltage, which varies approximately sinusoidally with the external cavity length *L*_ext_ under weak feedback, as observed in Fig. 2(a). Whilst analysis based on the L‒K equations is applicable only in the case of single-mode operation, models including the effects of multiple longitudinal modes have also been developed^1, 37^. In fact, under the regime of weak OF, the LFI signal can be approximated as a linear combination of signals arising from individual longitudinal modes of the solitary laser (see Methods)^38, 39^ in an analogous fashion to the interferogram recorded in Fourier transform spectroscopy. Crucially, this provides a means to recover the emission spectrum of the source over a wide spectral bandwidth, through Fourier analysis of the LFI voltage signal recorded under extension of the external cavity. Figure 2(b) shows an interferogram recorded at a drive current *I*_d_ = 950 mA, for which the laser operates in multiple longitudinal modes. In this case, multiple periodicities can be observed in the measured voltage signal, corresponding to multiple frequencies propagating in the cavity that each resonate at different external cavity lengths, as described by Eq. (4).

**Figure 3:**
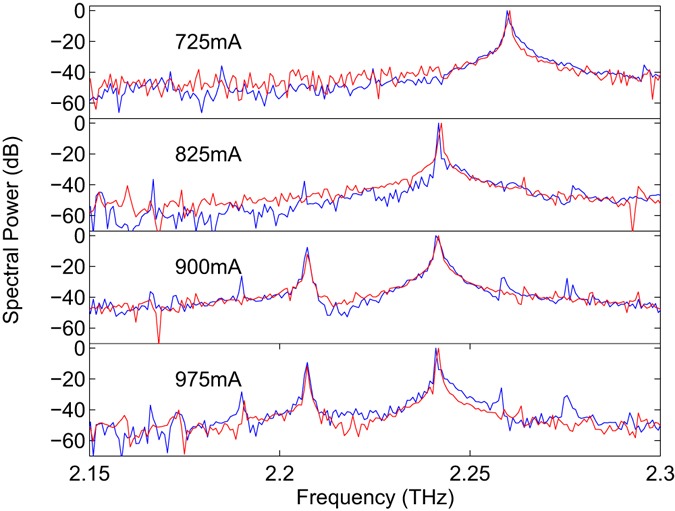
Normalised emission spectra obtained by laser-feedback interferometry (blue) and FTIR spectroscopy (red) for different dc driving currents, corresponding to both single- and multiple-mode operating regimes. The LFI spectra reveal lasing modes at 2.190 THz, 2.207 THz, 2.241 THz, 2.258 THz and 2.275 THz. In both cases the spectral resolution is 750 MHz.

To recover the spectral emission of the QCL a fast Fourier transform (FFT) of the LFI voltage signal was performed. For a cavity extension Δ*L*_ext_ = 200 mm the resulting spectral resolution of the FFT is *c*/2Δ*L*_ext_ = 750 MHz. We note, however, that LFI has been demonstrated previously in a THz QCL over an external path length of >10 m^40^. As such the spectral resolution of our experiment could, in principle, be increased beyond 15 MHz. Figure 2(c) and (d) show the normalised FFTs of the complete interferograms corresponding to data presented in Fig. 2(a) and (b), revealing the expected single- and multiple-longitudinal mode emission, respectively. From this data, a longitudinal mode spacing of Δ*v*_FP_ = ~17 GHz is obtained, in agreement with that expected for a laser cavity length *L*_c_ = 2.3 mm and active region effective refractive index *n* = 3.8 using the relation ${\rm{\Delta }}{\nu }_{{\rm{FP}}}=c/2n{L}_{{\rm{c}}}$. Figure 3 shows emission spectra recorded in this way for a range of laser driving currents, and plotted on a logarithmic scale to illustrate the dynamic range of the laser feedback-interferometer. The noise floor in our system is dominated by voltage noise at the input of the digital acquisition board, which is measured to be ~10 µV/√Hz. It is also worth noting that the response of lasers under weak OF is typically greatest just above threshold and rolls off with increasing driving current^25^; in our system, voltage signal amplitudes up to ~40 mV were recorded after amplification. As can be seen in Fig. 3, switching of the dominant mode from 2.258 THz to 2.241 THz is observed at low drive currents, with multiple-mode emission dominating at larger drive currents, as is typical behaviour in QCLs.

In order to validate these spectral characteristics obtained by LFI, spectral measurements were also performed using a conventional FTIR spectroscopy setup based on a Michelson interferometer [see Fig. 1 (box B) and Methods]. In this case radiation was collected from the rear facet of the laser with the front facet blocked to prevent feedback of radiation from the laser-feedback interferometer. Figure 3 shows comparative spectral data obtained for the same driving currents used for the LFI characterisation, at the same spectral resolution of 750 MHz (achieved with a single-trip path length extension of Δ*L*_FTIR_ = 200 mm). As can be seen, excellent agreement with the LFI characterisation is obtained in terms of both the absolute frequency of lasing modes as well as the overall spectral features recovered.

Interestingly, it can also be observed that the LFI characterisation reveals weak lasing modes, with spectral powers ~−30 dB relative to the dominant mode, that are not immediately apparent in the FTIR spectrometer data, for example modes at 2.190 THz, 2.258 THz and 2.275 THz observed for *I*_d_ ≥ 900 mA. Whilst such effects could, in general, occur due to the presence of OF in the laser-feedback interferometer, this is not expected in our case due to operating in the regime of weak OF. In fact, we have confirmed the presence of these lasing modes through FTIR measurements, but only under certain limited ranges of the interferometer path length (see Supplementary Information, Fig. S2). We therefore attribute this phenomenon to a propagation efficiency through our FTIR spectrometer that is highly sensitive to the alignment of the laser, which arises from the long optical path length (208 cm) from source to detector, coupled with the complex and frequency-dependent far-field beam pattern of the THz QCL. As has been described elsewhere^41–46^ the radiation patterns of THz QCLs with surface plasmon ridge waveguides are commonly characterised by multiple emission lobes, arising due to diffraction from both the laser facet and substrate^43–45^, as well as prominent ring-like interference fringes in the far-field. Several interpretations of these rings have been proposed, including aperture-like diffraction of the waveguide mode^43^, interference effects arising from reflections from the cryostat windows^44, 46^ and laser submount^46^, and interference effects that can be understood using a wire laser model that treats the QCL as a longitudinally-distributed source^41, 42, 47^. Crucially, the angular direction of these interference fringes relative to the cavity axis is determined by the radiation wavelength and thus differs for different longitudinal cavity modes^46^. It can therefore be expected that different longitudinal modes will propagate through our FTIR spectrometer with varying (frequency-dependent) coupling efficiencies to the detector entrance aperture, such that some modes can effectively be spatially filtered from the recorded spectrum. Indeed, this phenomenon of mode suppression in spectra recovered from our FTIR spectrometer has been confirmed experimentally (see Supplementary Information Fig. S1). Furthermore, this investigation confirms the presence of the weak laser modes that appear in the LFI spectra (at 2.190 THz, 2.258 THz and 2.275 THz, for example), although the mode intensities recovered using the FTIR spectrometer are found to depend strongly on the specific alignment conditions, with almost complete mode suppression occurring in some cases. In contrast to the FTIR approach, the self-aligning nature of our LFI scheme appears to reveal the full spectral content of the laser emission consistently. It is also worth noting that our LFI approach offers advantages in terms of measuring emission spectra when the laser is operating close to threshold. In such cases the output power, and hence the signal recovered by FTIR spectroscopy, is expected to be low. This is in contrast to the signal levels recorded by LFI, which are typically largest close to threshold^25, 48^.

### Frequency perturbation under optical feedback

As described by the L–K formalism, the modification to the laser carrier population under OF that is responsible for the voltage signal exploited in our LFI approach also induces a perturbation to the laser frequency, *v*. This effect is encapsulated through the transcendental *excess phase equation* [see Eq. (1)], which predicts that the laser frequency under OF is perturbed from that of the solitary laser *v*_0_ by an amount dependent on the external cavity round-trip time ${\tau }_{{\rm{ext}}}$, but within the bounds ${\rm{\Delta }}\nu ={\nu }_{0}-\nu =\pm \,C/2\pi {\tau }_{{\rm{ext}}}$. Crucially to our interferometric approach, ${\rm{\Delta }}\nu$ is small for weak feedback (*C* < 1) and long cavity lengths (${\tau }_{{\rm{ext}}}\gg 1/{\nu }_{0}$), such that the recovered spectral components of the laser emission deviate from those of the solitary laser by less than the 750 MHz spectral resolution limit of the LFI measurement as determined by the cavity extension ${\rm{\Delta }}{L}_{{\rm{ext}}}$.

**Figure 4:**
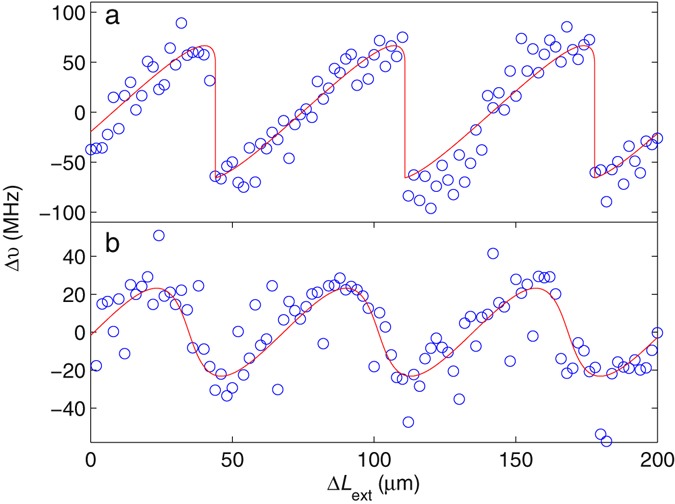
Frequency perturbation Δ*v* of the laser cavity mode at ~2.241 THz under optical feedback, measured over a cavity extension in the laser-feedback interferometer Δ*L*_ext_ = 200 µm, for feedback parameters (**a**) *C* ≈ 1.5 and (**b**) *C* ≈ 0.5 (blue circles). Also shown are fits to the excess phase equation, Eq. (1) (red lines).

In order to confirm the frequency perturbation of individual laser modes under OF, spectra were recorded using the FTIR spectrometer for a series of external cavity lengths in the laser-feedback interferometer and for controlled levels of OF to the laser (see Methods). In this case a long path length extension of Δ*L*_FTIR_ = 600 mm was used to attain a higher spectral resolution from the FTIR spectrometer. Figure 4(a) shows the frequency perturbation of the laser cavity mode at ~2.241 THz, measured over a cavity extension in the laser-feedback interferometer Δ*L*_ext_ = 200 µm, for a feedback parameter *C* ≈ 1.5. Under this moderate feedback the frequency of the laser mode *v* varies almost linearly with ${L}_{{\rm{ext}}}$ over a cavity extension ${\rm{\Delta }}{L}_{{\rm{ext}}}\approx c/2{\nu }_{0}$ before repeating this trend in subsequent LFI fringes (which are also spaced by ${\rm{\Delta }}{L}_{{\rm{ext}}}=c/2{\nu }_{0}$). Figure 4(b) shows the equivalent behaviour under weak OF, with *C* ≈ 0.5. In this case the laser frequency oscillates approximately sinusoidally as a function of ${L}_{{\rm{ext}}}$, and with a smaller amplitude of perturbation. It is important to note that the laser emission is not dominated by external cavity modes in this experiment; rather, the perturbation to the laser carrier population under OF causes a periodic perturbation of the laser frequency with varying ${L}_{{\rm{ext}}}$, as described by the excess phase equation [Eq. (1)], which serves to ensure matched phases for the outgoing and returning field at the laser facet. To validate this description, the data was fitted to Eq. (1) with *C* treated as a free parameter. For this fitting procedure the linewidth enhancement factor *α* = 0 was assumed, as is typical for intersubband lasers for which the electron subbands have parallel curvature in k-space^31, 49^. As is evident from Fig. 4, the excess phase equation reproduces the data remarkably well in both cases, in terms of both of the dependence on ${L}_{{\rm{ext}}}$ and the maximum amplitude of the measured frequency perturbation. From our measurements, it is also possible to analyse the frequency perturbation of the weaker mode at ~2.275 THz. In this case, similar results are obtained, revealing an amplitude of perturbation of ±~100 MHz for *C* ≈ 1.5. Indeed, this similar behaviour for different cavity modes is expected from the excess phase equation [Eq. (1)], which predicts that the maximum frequency perturbation under OF depends only on the feedback parameter *C* and the external cavity round-trip time ${\tau }_{{\rm{ext}}}$, and is independent of the mode frequency.

**Figure 5:**
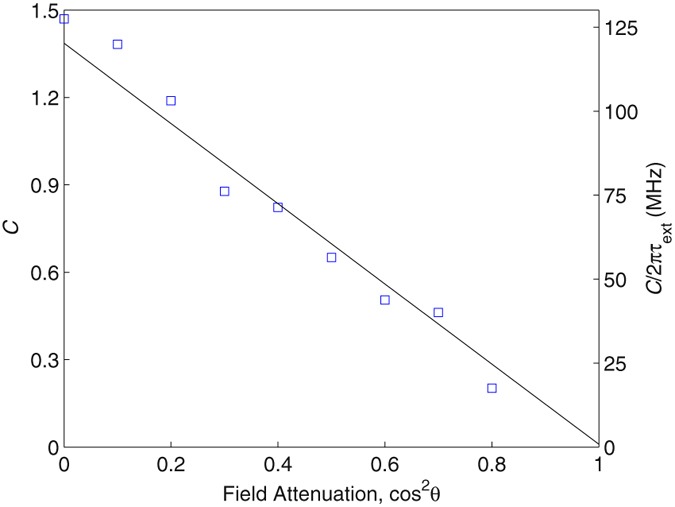
Feedback parameter *C* (left axis) obtained from fitting the excess phase equation to measurements of the frequency perturbation Δ*v* of the laser cavity mode at ~2.241 THz under optical feedback, for different levels of field attenuation in the external cavity. A linear fit to this data is shown (solid line). Also plotted (right axis) is the maximum amplitude of frequency perturbation *C*/2π*τ*_ext_ determined from these fits.

Figure 5 shows the feedback parameter obtained from this fitting procedure for varying degrees of field attenuation in the external cavity. The double-pass field attenuation factor plotted here is calculated from cos^2^ *θ*. A linear relationship is obtained, in agreement with the expected proportionality between *C* and $\sqrt{{R}_{{\rm{ext}}}}$ [see Eq. (3)]. Also plotted is the maximum amplitude of frequency perturbation ${C}/2\pi {\tau }_{{\rm{ext}}}$ determined from these fits. Importantly, the perturbation ${\rm{\Delta }}\nu$ remains smaller than the spectral resolution limits of our LFI measurement for all levels of OF achievable within our setup, such that these effects have negligible impact on the spectral data recovered using our LFI approach.

## Conclusions

In summary, we have reported a new modality of laser-feedback interferometry in which changes to the terminal voltage of a semiconductor laser in response to an extension of the external cavity are used to infer the emission spectrum of the solitary laser. Whilst applicable across all types of semiconductor lasers, our scheme offers a simple alternative to Fourier transform infrared spectroscopy approaches typically employed for spectral characterisation of lasers operating in the mid- and far-infrared spectral region. We have demonstrated this approach using a terahertz-frequency quantum cascade laser source, for both single- and multiple-longitudinal mode operating regimes, and were able to resolve spectral features not reliably resolved using traditional FTIR spectroscopy. In this respect, our approach offers a notable advantage compared to FTIR-based approaches to the spectral characterisation of THz QCLs. We have also reported the first measurement, to our knowledge, of the frequency perturbation of solitary laser modes under different levels of optical feedback, and found excellent agreement with predictions of the excess phase equation central to the theory of lasers under optical feedback.

## Methods

### Laser response to optical feedback

The response of a laser subject to OF can be described using the well-established rate equation model for the complex field and carrier density proposed by Lang and Kobayashi, which includes the influence of feedback through a time-delayed field term^36^. Under the steady-state condition these rate equations reduce to a set of equations for the laser frequency $\nu$ and the threshold carrier density *n*^1, 9^:

1 $$2\pi {\tau }_{{\rm{ext}}}({\nu }_{0}-\nu )=C\,\sin (2\pi \nu {\tau }_{{\rm{ext}}}+\arctan (\alpha )),$$

2 $$n-{n}_{0}=-\,\tilde{\beta }\,\cos (2\pi \nu {\tau }_{{\rm{ext}}})$$

where the subscript 0 indicates values for the solitary laser without feedback, $\alpha$ is the linewidth enhancement factor, $\tilde{\beta }$ represents the coupling rate of feedback relative to the rate of carrier density gain, and ${\tau }_{{\rm{ext}}}$ is the round-trip delay in the external cavity given by ${\tau }_{{\rm{ext}}}=2{L}_{{\rm{ext}}}/c$, in which ${L}_{{\rm{ext}}}$ is the external cavity length and *c* is the speed of light in the external cavity. The dimensionless feedback parameter *C* is defined as

3 $$C=\varepsilon \frac{{\tau }_{{\rm{ext}}}}{{\tau }_{{\rm{L}}}}\sqrt{1+{\alpha }^{2}}\sqrt{\frac{{R}_{{\rm{ext}}}}{{R}_{{\rm{L}}}}}(1-{R}_{{\rm{L}}})$$

in which ${R}_{{\rm{ext}}}$ is the reflectivity of the external cavity mirror, ${R}_{{\rm{L}}}$ is the reflectivity of the emitting laser facet, ${\tau }_{{\rm{L}}}$ is the round-trip time for light in the laser cavity, and $\varepsilon$ is the fraction of the reflected light coupled coherently to the laser mode that accounts for loss due to attenuation in the external cavity, spatial mode mismatch between the reflected and the cavity mode, and other optical losses. Per the transcendental *excess phase equation* [Eq. (1)], the perturbed laser frequency under OF is close to that of the solitary laser for weak feedback (C < 1), such that $\nu \approx {\nu }_{0}$. Eq. 2 elucidates the form of the LFI signal recorded via the perturbation induced on the laser voltage or the emitted power under OF, both of which can be approximated as being proportional to the change in carrier density for small perturbations^2^.

This model based on the L‒K formalism inherently assumes lasing in a single longitudinal mode, in which case the ac component of the linearized terminal voltage signal (the laser-feedback interferometry signal, ${V}_{{\rm{LFI}}}$) can be written as ${V}_{{\rm{LFI}}}=\beta \,\cos (2{\rm{\pi }}\nu {\tau }_{{\rm{ext}}})$, where amongst other factors $\beta$ is proportional to the rate at which optical feedback is coupled back into the laser cavity^50^. Nevertheless, for the case of a laser emitting on $N$ longitudinal modes, the LFI signal can be approximated as a linear combination of signals arising from individual modes^38, 39^. In this case, the ac component of the linearized terminal voltage signal can be expressed, in the case of weak feedback, as

4 $${V}_{{\rm{LFI}}}=\sum _{i=1}^{N}{\beta }_{i}\,\cos (2\pi {\nu }_{i}{\tau }_{{\rm{ext}}})$$

where ${\nu }_{i}$ is the frequency of the *i*^th^ mode of the solitary laser and ${\beta }_{i}$ is a prefactor relating to the intensity of that mode (through the rate at which optical feedback is coupled back into it).

### Quantum cascade laser source

The THz QCL consisted of a 14 μm-thick bound-to-continuum active region^51^ emitting at ~2.25 THz (*~*133 μm), which was processed into a semi-insulating surface-plasmon ridge waveguide with dimensions of 2.3 mm × 200 μm. The device was cooled using a continuous-flow helium cryostat and maintained at a heatsink temperature of 25 K. At this temperature, the threshold current was 700 mA, and the device emitted a maximum power ~2.5 mW.

### Spectral characterisation using laser-feedback interferometry

The emission from the laser was collimated using a 2-inch diameter *f*/2 off-axis parabolic reflector and reinjected to the laser cavity using a flat planar mirror. Two wire-grid polarisers were positioned in the collimated beam path, with the first oriented parallel to the major axis of the elliptically polarised emission (i.e. parallel to the growth direction of the QCL heterostructure), and the second orientated at an angle *θ* relative to the axis of the first to control the level of feedback to the laser. Interferograms were recorded by extending the external cavity over a distance Δ*L*_ext_ = 200 mm, from a cavity length *L*_ext_ = 0.52 to 0.72 m, using a computer-controlled translation stage. The corresponding change in the QCL terminal voltage due to feedback, *V*_LFI_, was amplified using a 22 dB ac-coupled differential voltage amplifier and recorded at a sampling rate of 8 kHz (corresponding to 2 µm increments of the mirror position) using a 14-bit digital acquisition board. To improve the signal-to-noise level of the system, ten measurements were averaged for each interferogram recorded. For these measurements, the rear facet of the laser was blocked to prevent any feedback of radiation from the FTIR spectrometer. No purging of the system was used.

### Spectral characterisation using FTIR spectrometer

Emission from the rear facet of the QCL was collected using a 2-inch diameter *f*/2 off-axis parabolic reflector, and coupled to a standard Michelson interferometer arrangement employing a silicon beam splitter and helium-cooled germanium bolometer (see Fig. 1, dashed box). The planar mirror in the variable arm of the interferometer was translated over a distance of up to Δ*L*_FTIR_ = 600 mm using a computer-controlled translation stage, and the corresponding detector signal was recorded at a sampling rate of 2.67 kHz (corresponding to 4 times the Nyquist frequency) using a 14 bit digital acquisition board. The total optical path length from QCL to detector was 208 cm at the furthest extension of the mirror. An isolator consisting of a polariser and quarter-wave plate were employed to prevent optical feedback to the laser from the FTIR spectrometer. No purging of the system was used. The noise floor in this system is dominated by voltage noise at the input of the digital acquisition board (~10 µV/√Hz), with the signal amplitude scaling proportionally with emitted power, up to a maximum value ~1.4 V (at a drive current *I*_d_ = 875 mA).

### Measurement of frequency perturbation under optical feedback

The external cavity length in the laser-feedback interferometer was extended from *L*_ext_ = 0.6198 to 0.6200 m in increments of 2 µm, and a single FTIR measurement performed at each step using radiation coupled from the rear facet of the laser. In order to prevent coupling of radiation between the LFI and FTIR systems, the polariser in the FTIR spectrometer was oriented orthogonal to the first polariser in the laser-feedback interferometer (i.e. orthogonal to the major axis of the elliptically polarised laser emission). Measurements were repeated with varying levels of OF in the range *C* ≈ 0 to *C* ≈ 1.5, controlled through rotation of the second wire grid polariser. For these measurements, a driving current *I*_d_ = 825 mA was used. The precise frequency of the laser cavity mode at ~2.241 THz was extracted from the FTIR spectra by fitting the absolute magnitude of a cardinal sine (sinc) function and taking the centroid frequency.

### Additional Data

Additional data sets related to this publication are available from the University of Leeds data repository at https://doi.org/10.5518/138.

## Electronic supplementary material

Supplementary information
