# Publisher Correction: Measurement of the emission spectrum of a semiconductor laser using laser-feedback interferometry

**DOI:** 10.1038/s41598-018-36015-w

**Published:** 2018-11-30

**Authors:** James Keeley, Joshua Freeman, Karl Bertling, Yah Leng Lim, Reshma A. Mohandas, Thomas Taimre, Lianhe H. Li, Dragan Indjin, Aleksandar D. Rakić, Edmund H. Linfield, A. Giles Davies, Paul Dean

**Affiliations:** 1 School of Electronic and Electrical Engineering, University of Leeds, Leeds, LS2 9JT UK; 2 School of Information Technology and Electrical Engineering, The University of Queensland, St Lucia, QLD 4072 Australia; 3 School of Mathematics and Physics, The University of Queensland, St Lucia, QLD 4072 Australia

Correction to: *Scientific Reports* 10.1038/s41598-017-07432-0, published online 03 August 2017

The original version of this Article contained a typographical error in the spelling of the author Yah Leng Lim, which was incorrectly given as Yah L. Lim. This has now been corrected in the PDF and HTML versions of the Article.

