# Supplementary material for: Measurement of the emission spectrum of a semiconductor laser using laser-feedback interferometry
Source: Sci Rep. 2017 Aug 3;7:7236. doi: 10.1038/s41598-017-07432-0 (PMC5543105; doi:10.1038/s41598-017-07432-0)
Supplement: Supplementary file 1 — Supplementary information [file 41598_2017_7432_MOESM1_ESM.pdf]

Measurement of the emission spectrum of a semiconductor laser using laser-feedback interferometry

James Keeley,[1] Joshua Freeman,[1] Karl Bertling,[2] Yah Leng Lim,[2] Reshma A. Mohandas,[1] Thomas Taimre,[3] Lianhe H. Li,[1] Dragan Indjin,[1] Aleksandar D. Rakić,[2] Edmund H. Linfield,[1] A. Giles Davies[1] and Paul Dean[1*]

1 School of Electronic and Electrical Engineering, University of Leeds, Leeds, LS2 9JT, UK
2 School of Information Technology and Electrical Engineering, The University of Queensland, St Lucia, QLD 4072, Australia
3 School of Mathematics and Physics, The University of Queensland, St Lucia, QLD 4072, Australia
*p.dean@leeds.ac.uk

The beam propagation efficiency through our Fourier transform infrared (FTIR) spectrometer has been found to be highly sensitive to the alignment of the laser and spectrometer, which is attributed principally to the long optical path length (208 cm) from source to detector. Furthermore, the radiation patterns of terahertz quantum cascade lasers (QCLs) with surface plasmon ridge waveguides are known to exhibit prominent ring-like interference fringes in the far-field, which have been interpreted in terms of aperture-like diffraction of the waveguide mode [1], interference effects arising from reflections from the cryostat windows [2, 3] and laser submount [3], and interference effects that can be understood using a wire laser model that treats the QCL as a longitudinally-distributed source [4, 5, 6]. Crucially, the angular direction of these fringes depends on the frequency of individual laser modes [3]. As such, different longitudinal modes can be expected to propagate through our FTIR spectrometer with varying (frequency-dependent) coupling efficiencies to the detector entrance aperture, such that some modes can effectively be spatially filtered from the recorded spectrum.

To demonstrate this phenomenon, an interferogram was recorded for a long path length extension of the interferometer $\Delta L_{\mathrm{FTIR}} =$ 600 mm, as shown in Fig. S1(a). Figure S1(b) shows a map of the laser spectra obtained by performing fast Fourier transforms of subsets of this data, isolated using a moving window of width $\Delta L_{\mathrm{FTIR}} =$ 75 mm. As can be observed, in addition to the main lasing mode at 2.241 THz, weaker modes at 2.207 THz, 2.258 THz, and 2.275 THz are also apparent. However, these modes are not necessarily resolved simultaneously and the recovered relative mode intensities are found to depend strongly on the region of the interferogram selected, with complete mode suppression occurring over certain regions. This effect is explained by poor coupling of these modes to the detector for certain ranges of the interferometer optical path length. Similar effects were also observed when the laser was misaligned in a direction perpendicular to the optical axis of the FTIR, with additional weaker lasing modes becoming observable under only specific alignment conditions.

One consequence of this phenomenon is the unreliable representation of lasing spectra obtained by our FTIR spectrometer, particularly in the case of high-resolution spectra for which a long path length extension is necessary. Figure S2 compares exemplar spectra obtained using both laser-feedback interferometry (LFI) and our FTIR spectrometer, for a laser driving current $I_{\mathrm{d}} =$ 900 mA. In this case a narrow window of width $\Delta L_{\mathrm{FTIR}} =$ 50 mm, centred at $\Delta L_{\mathrm{FTIR}} =$ 573 mm, has been applied to the FTIR data in order to illustrate the presence of weaker lasing modes other than the dominant modes at 2.207 THz and 2.241 THz. The LFI spectrum plotted in this figure corresponds to the data presented in Fig. 3 of the manuscript, but with this same narrow window applied, which has the effect of reducing the spectral resolution to 3 GHz. It can be observed that the weaker lasing modes at 2.190 THz, 2.258 THz and 2.275 THz, which are resolved by the LFI measurement but are not apparent in the high-resolution FTIR data presented in Fig.3 of the manuscript, are also represented in the FTIR data under these conditions.

Fig. S1 – (a) Interferometric fringes recorded using a FTIR spectrometer based on a Michelson interferometer arrangement employing a silicon beam splitter and helium-cooled germanium bolometer (see main manuscript for details). A laser driving current $I_{\mathrm{d}} = $ 925 mA was used. (b) Colour map of the normalised laser emission spectra obtained by performing fast Fourier transforms of subsets of the data presented in (a), isolated using a moving window of width $\Delta L_{\mathbf{FTIR}} = $ 75 mm. The *x*-axis corresponds to the position of the centre of this window.

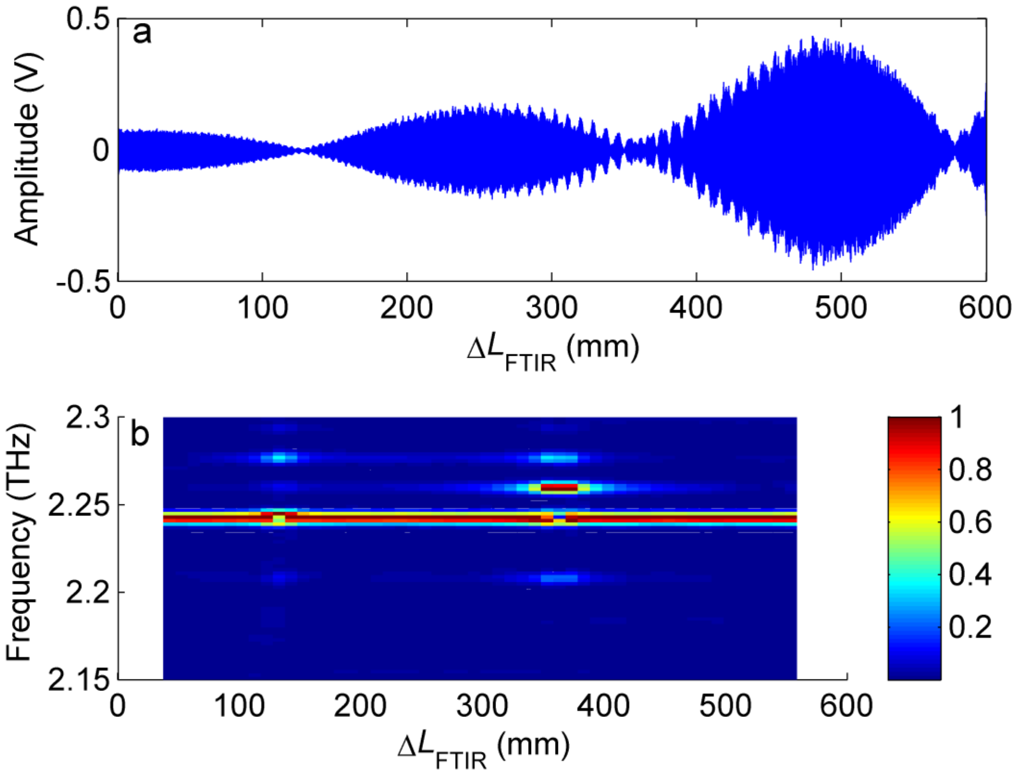

Fig. S2 – Normalised emission spectra obtained by laser-feedback interferometry (blue) and FTIR spectroscopy (red) for a laser driving current $I_d = 900$ mA. Both spectra reveal lasing modes at 2.190 THz, 2.207 THz, 2.241 THz, 2.258 THz and 2.275 THz, indicated by arrows. In both cases the spectral resolution is 3 GHz.

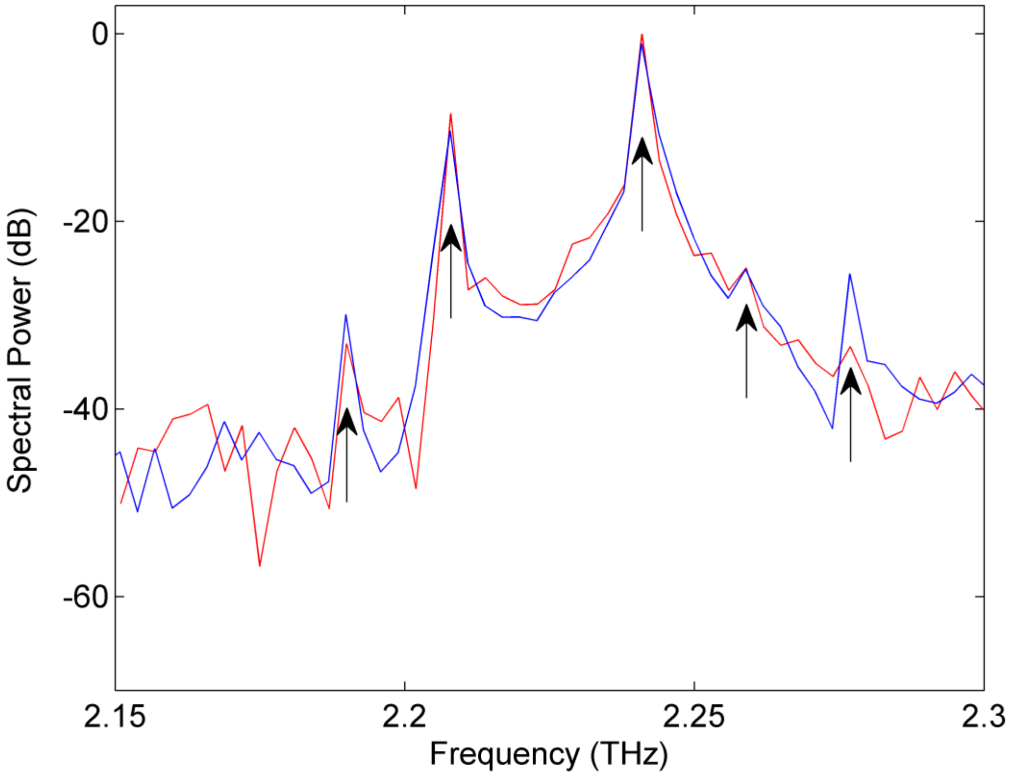

References

1. Hübers, H.-W. *et al.*, Terahertz quantum cascade laser as local oscillator in a heterodyne receiver. *Optics Express* **13** (15), 5890-5896 (2005).
2. Bründermann, E. *et al.*, Turn-key compact high temperature terahertz quantum cascade lasers. *Optics Express* **14** (5), 1829-1841 (2006).
3. Röben, B., Wienold, M., Schrottke, L. & Grahn, H. T., Multiple lobes in the far-field distribution of terahertz quantum-cascade lasers due to self-interference. *AIP Advances* **6**, 065104 (2016).
4. Hajenius, M. *et al.*, Surface plasmon quantum cascade lasers as terahertz local oscillators. *Optics Letters* **33** (4), 312-314 (2008).
5. Salih, M. *et al.*, Terahertz quantum cascade lasers with thin resonant-phonon depopulation active regions and surface-plasmon waveguides. *Journal of Applied Physics* **113** (11), 113110 (2013).
6. Orlova, E. E. *et al.*, Antenna Model for Wire Lasers. *Physical Review Letters* **96** (17), 172904 (2006).
